# Some Ultra-Processed Foods Are Needed for Nutrient Adequate Diets: Linear Programming Analyses of the Seattle Obesity Study

**DOI:** 10.3390/nu13113838

**Published:** 2021-10-28

**Authors:** Skyler Hallinan, Chelsea Rose, James Buszkiewicz, Adam Drewnowski

**Affiliations:** 1 Paul G. Allen School of Computer Science & Engineering, University of Washington, Seattle, WA 98105, USA; 2 Center for Public Health Nutrition, University of Washington, Seattle, WA 98105, USA; cmr013@uw.edu (C.R.); buszkiew@uw.edu (J.B.); adamdrew@uw.edu (A.D.); 3 Department of Epidemiology, University of Washington, Seattle, WA 98105, USA

**Keywords:** food patterns, diet quality, ultra-processed foods, linear programming, SOS III

## Abstract

Typical diets include an assortment of unprocessed, processed, and ultra-processed foods, along with culinary ingredients. Linear programming (LP) can be used to generate nutritionally adequate food patterns that meet pre-defined nutrient guidelines. The present LP models were set to satisfy 22 nutrient standards, while minimizing deviation from the mean observed diet of the Seattle Obesity Study (SOS III) sample. Component foods from the Fred Hutch food frequency questionnaire comprised the market basket. LP models generated optimized 2000 kcal food patterns by selecting from all foods, unprocessed foods only, ultra-processed foods only, or some other combination. Optimized patterns created using all foods contained less fat, sugar, and salt, and more vegetables compared to the SOS III mean. Ultra-processed foods were the main sources of added sugar, saturated fat and sodium. Ultra-processed foods also contributed most vitamin E, thiamin, niacin, folate, and calcium, and were the main sources of plant protein. LP models failed to create optimal diets using unprocessed foods only and ultra-processed foods only: no mathematical solution was obtained. Relaxing the vitamin D criterion led to optimized diets based on unprocessed or ultra-processed foods only. However, food patterns created using unprocessed foods were significantly more expensive compared to those created using foods in the ultra-processed category. This work demonstrates that foods from all NOVA categories can contribute to a nutritionally adequate diet.

## 1. Introduction

The NOVA food classification scheme [1] assigns foods into four categories: ultra-processed, processed, unprocessed, and culinary ingredients. Accounting for >60% of energy in the American diet [2,3], ultra-processed foods have been linked to a variety of adverse health outcomes, including obesity [4], diabetes [5], hypertension [6], depression [7], cancer [8], and all-cause mortality [9]. There is also an economic dimension [10,11]. In the Seattle Obesity Study III (SOS III) [12], percent energy from ultra-processed foods was associated with lower diet quality but also with significantly lower food spending [10,11]. Study participants in the bottom decile of estimated diet cost ($216/person/month) derived 67.5% energy from ultra-processed foods as compared to 48.7% for those in the top decile ($370/person/month) [11]. The consumption of lower-cost ultra-processed foods depends on household socioeconomic status [10,11]. Unobserved socioeconomic factors may confound any observed relation between diet quality and health [13,14].

Ultra-processed foods are formally defined as industrial creations that contain chemical ingredients (flavors, stabilizers, or emulsifiers) not used in normal home cooking along with added fat, sugar, and salt [1]. Classed as ultra-processed are sugary beverages, sweets, desserts, pizza, and breakfast cereals, but also commercial yogurts, juices, and whole grain breads. Based on past analyses of 384 component foods of the Fred Hutch food frequency questionnaire, the ultra-processed classification captured not only fats and sweets (73%), the intended target, but virtually all grain foods (91%), along with most beans, nuts, and seeds (70%) [11]. Grains and cereals are rarely eaten raw; some degree of processing is usually involved. Assigned to the unprocessed category were most fruit, vegetables, meat, poultry, and fish [11].

Many foods classified as ultra-processed, including breads, ready to eat cereals, and some beverages are fortified with vitamins and minerals [15]. It is possible that incorporating a proportion of such foods in the habitual diet allows for micronutrient requirements to be met at a more affordable cost. The mathematical technique of linear programming (LP) permits the construction of theoretical dietary patterns that satisfy multiple nutrient requirements under a variety of constraints [16,17,18,19]. Such models can then be used to test the feasibility of proposed dietary guidance. For example, one LP study [20] tested whether a stringent sodium reduction goal (1500 mg/day at the time) was compatible with nutrient-adequate diets. Whereas the 2300 mg/d sodium goal was feasible, the more stringent 1500 mg/day sodium goal was incompatible with nutrient adequacy and no mathematical solution was obtained [20]. Modeled food patterns have also been created to minimize diet cost [21], or to minimize diet-associated greenhouse gas emissions [18].

This project sought to determine whether nutrient-adequate food patterns could be created using unprocessed foods only, or using ultra-processed foods only. The relative cost of the optimized food patterns was of special interest. Nutrient composition and cost data for component foods of the Fred Hutch FFQ served as input. All foods were assigned into NOVA categories. Observed dietary intakes for 857 male and female adults came from the Seattle Obesity Study III [12]. The goal was to create optimized 2000 kcal/d food patterns that met standards for 22 nutrients while respecting existing food habits of SOS III participants.

## 2. Materials and Methods

### 2.1. Study Design and Participants

The SOS III was a population-based study of adult men and women living in King, Pierce, and Yakima Counties in Washington State [12]. County-specific recruitment schemes used address-based sampling stratified by residential property values along with community outreach to ensure broad representation by socioeconomic status and race/ethnicity. Recruitment and in-person data collection were conducted from July 2016–May 2017 by local staff at each site.

Eligible participants were aged 21–59 years, were principal household food shoppers, without any mobility issues, and not pregnant or breastfeeding. Written consent was provided during the in-person visit before starting the study procedures. Data were collected in English and Spanish (in Yakima County). All study procedures were approved by the Institutional Review Boards (IRBs) of respective sites. For analyses, participants were excluded due to implausible caloric intakes: 12 participants with kcal >5000 and 3 participants with kcal <500. The present analytical sample was based on 857 male and female respondents.

### 2.2. FFQ Component Foods

The Fred Hutch Food Frequency Questionnaire (FFQ) was used to collect dietary intake data [22]. The FFQ consists of a list of 126 line-item foods and 384 component foods that are a part of the FFQ structure but are not visible to the respondent. Each food’s energy and nutrient content are calculated based on a weighted average of component foods. A nutrient database containing the nutrient content of each of the 384 component foods was derived from the USDA Food and Nutrient Database for Dietary Studies [10,23,24,25]. For the present calculations, we omitted drinking water, tea, coffee, diet soda, beer, wine and whiskey. The 360 unique foods were then aggregated into seven major food groups: 1. fats/sugary beverages/non-grain sweets; 2. dairy; 3. fruits and fruit juices; 4. vegetables; 5. beans/nuts/seeds; 6. grains; 7. meats/poultry/fish.

The same foods were also assigned to the four NOVA food processing categories as shown in Table 1. Fresh, dry, or frozen foods that had been subjected to minimal or no processing were treated as unprocessed. These included fresh meat, fish, fruits (such as apple, banana, apricots), salad, milk, vegetables (broccoli, green beans, potato), eggs, legumes, and unsalted nuts (raisins and prunes) and seeds. Culinary ingredients were sugar, animal fats (butter) and oils (olive oil, canola oil, corn oil), and salt (21). Based on published NOVA criteria, the addition of fat, sugar and salt to wholesome fresh foods transformed them into processed foods. Among processed foods were cheese, ham, canned fruits, and canned beans. Among FFQ component foods that were classified as ultra-processed were commercial breads, jams and jelly, ready to eat and other breakfast cereals, sweet snacks (cookies and cakes), pizza, potato chips or tortilla chips, soft drinks (sodas and fruit drinks), French fries, sauces (ketchup, mayonnaise), desserts (ice cream, frozen yogurt, sherbet), fast food meals, juices and soups.

### 2.3. Nutrient Recommendations

Reference daily values (DVs) were based on the US Food and Drug Administration (FDA) and other standards. The reference amounts were: protein (50 g), fiber (28 g), vitamin A (3000 IU), vitamin C (90 mg), vitamin D (20 mcg), calcium (1300 mg), iron (18 mg), potassium (4700 mg) and magnesium (420 mg). The maximum recommended values (MRVs) for the LIM component were: added sugar (50 g), saturated fat (20 g) and sodium (2300 mg).

### 2.4. Energy and Nutrient Intakes and Estimated Diet Cost

Estimates of individual-level daily diet cost were obtained by joining dietary intake data with county specific retail prices for 360 FFQ component foods. Retail prices were obtained from large supermarkets in King, Pierce and Yakima counties following standard and published procedures [10,11]. Retail prices converted to dollars per 100 g edible portion were added to the nutrient database, to parallel nutrient values, expressed as amounts (g/mg/IU) per 100 g edible portion. In this way, each of the 360 foods in the nutrient database was associated with 45 nutrient vectors and a single cost vector. The procedures of estimating diet costs from FFQ have been described previously [26]. The calculated cost of each modeled food pattern was expressed per 2000 kcal/d.

### 2.5. Linear Programming to Generate a Nutrient-Adequate Diet

Linear programming (LP) models used to optimize dietary patterns have an objective function, a set of nutritional goals, and a set of consumption constraints. The objective function typically measures the deviation from current eating behaviors as the model seeks a nutritionally adequate diet. Foods in different amounts are then selected from the market basket to create a food pattern that satisfies a minimum set—or an optimal range—of nutrient goals [16].

The linear objective function can vary depending on study purpose: some studies have minimized the deviation from current eating behaviors [17,18,19,20,27,28,29], while other studies have maximized or minimized total energy [30] or minimized cost [16]. Table 2 shows observed data from SOS III for the entire adult sample (n=857). The 22 unique nutrient constraints that needed to be met are also shown in Table 2. We label each of these nutrients from 1 to 22 in the order displayed in this table.

#### 2.5.1. Constructing the Objective Function

To develop our linear program, we first defined the following variables. We use the keywords “observed” and “optimized” to refer to quantities from our dataset and from our linear program respectively. The observed quantities were computed from *averaging* consumption over all participants in the SOS III dataset after excluding select outliers.

(1) $$\begin{array}{cc} \mathrm{For}\;i=1\;\mathrm{to}\;7 & \;\mathrm{and}\;j=1\;\mathrm{to}\;22,\;\mathrm{let}: \end{array}$$

(2) $$\begin{array}{cc} g_{o_{i}} & =\mathrm{observed}\;\mathrm{consumption}\;\left(g\right)\;\mathrm{of}\;\mathrm{food}\;\mathrm{group}\;i \end{array}$$

(3) $$\begin{array}{cc} g_{p_{i}} & =\mathrm{optimized}\;\mathrm{consumption}\;\left(g\right)\;\mathrm{of}\;\mathrm{food}\;\mathrm{group}\;i \end{array}$$

(4) $$\begin{array}{cc} n_{o_{i_{j}}} & =\mathrm{observed}\;\mathrm{consumption}\;\mathrm{of}\;\mathrm{food}\;\mathrm{group}\;i\;\mathrm{and}\;\mathrm{nutrient}\;j \end{array}$$

(5) $$\begin{array}{cc} n_{p_{i_{j}}} & =\mathrm{optimized}\;\mathrm{consumption}\;\mathrm{of}\;\mathrm{food}\;\mathrm{group}\;i\;\mathrm{and}\;\mathrm{nutrient}\;j \end{array}$$

The objective function is important and helps govern the solution output from the model. We defined the following three objective functions:

##### Grams Objective Function

This objective function minimizes the relative difference between the observed and optimized gram quantities from each of the food groups. Formally, this is defined as:

$$\begin{array}{c} \mathrm{Minimize}\;\sum_{i=1}^{7}\frac{|g_{o_{i}}-g_{p_{i}}|}{g_{o_{i}}}. \end{array}$$

This has been commonly utilized in previous studies to ensure that the model-generated diet has food group weights that are similar to the average diet. However, the total weight of foods in each food group may not assure optimal nutrient distribution, since each major food groups contains a variety of different food subgroups and categories. This may result in a model output that is much different from the SOS III mean.

##### Nutrient Objective Function

This objective function minimizes the relative difference between the observed and optimized nutrient quantities from each of the food groups. Formally, this is defined as:

$$\begin{array}{c} \mathrm{Minimize}\;\sum_{i=1}^{7}\sum_{i=1}^{22}\frac{|n_{o_{i_{j}}}-n_{p_{i_{j}}}|}{n_{o_{i_{j}}}}. \end{array}$$

This objective function is more precise than the grams objective function; by minimizing the nutrient differences in each food group, the model output using this objective function should be closer to the actual food basket consumed by the average person.

##### Grams and Nutrient Objective Function

This objective function combines the previous two objective functions, utilizing both nutrient and gram quantities. Formally, this is defined as:

$$\begin{array}{c} \mathrm{Minimize}\;\sum_{i=1}^{7}\left(\frac{|g_{o_{i}}-g_{p_{i}}|}{g_{o_{i}}}+\sum_{i=1}^{22}\frac{|n_{o_{i_{j}}}-n_{p_{i_{j}}}|}{n_{o_{i_{j}}}}\right). \end{array}$$

This objective function encapsulates information from the first two objective functions by combining them. This will result in a diet that is similar to the average consumed diet. We used this objective function in our modeling.

#### 2.5.2. Modeling

The objective function was supplemented with nutrient constraints on the modeled food patterns, as defined in Table 2. Thus, the output of the linear programming was a set of foods and their respective quantities that satisfied the nutritional requirements and optimized the current objective function.

We used linear programming to construct optimized diets under the three objective functions we identify above, including foods with certain processing levels. Table 3 lists the combinations of food processing levels attempted.

For each of these diets, we determined first if a solution was feasible; if it was not, then there was no way to create a nutrient adequate diet using the current market basked of Fred Hutch FFQ component foods. If a solution was found, we divided the objective function by 161, the number of items in our objective function summation, to determine the model’s average deviation from the SOS III diet.

## 3. Results

### 3.1. SOS III Observed Diets

We first describe the population data from the SOS III dataset in Table 4. The population was approximately evenly spread among age groups, and was primarily women. There were comparable proportions of non-Hispanic white and Hispanic people. Most of the population were college graduates or in higher education. Finally, household incomes were relatively uniformly distributed.

### 3.2. Feasibility of Alternative Models

Table 5 shows that LP solutions were obtained only under specific market basket conditions. First, an LP solution was obtained when the market basket contained foods from all four NOVA categories: ultra-processed, processed, unprocessed, and culinary ingredients. A solution was also obtained using a market basket that contained both processed and ultra-processed foods. However, in this analysis based on FFQ component foods, a market basket limited to only ultra-processed foods did not allow for the creation of nutrient adequate food patterns. Furthermore, limiting the market basket to unprocessed foods alone did not yield a mathematical solution. Nutrient adequate food patterns were only viable when both unprocessed and processed foods were included.

### 3.3. Model Outputs

Table 6 displays the nutrient composition of the three models that arrived at a mathematical solution and compares them to the SOS III average. Note that Models 2 and 3 produced the same output, so their results are condensed into one column.

Both models output a food pattern with exactly 2000 calories, similar to the average of 1953 from the SOS III. Energy density of the observed diet was 1.16 kcal/g. Energy density in Model 1 (all food groups) was 1.14 kcal/g. However, in Model 2, which excluded unprocessed foods, energy density was 1.51 kcal/g. Lower energy density has been used as a proxy indicator of higher diet quality.

The modeled food patterns were of higher quality than the observed diet of SOS III participants. All three models were slightly lower in the amounts of fats and sweets and of grains compared to the SOS III average. In addition, all three models contained a significantly smaller quantity of meat, poultry, and fish as compared to the observed SOS III diets. Whereas Model 1 significantly increased the amount of vegetables compared to the SOS III average, Models 2 & 3 significantly decreased the quantity of vegetables. A similar pattern emerged for milk and dairy: Model 1 and Models 2 & 3 increased and decreased the quantity of milk and dairy in comparison to the SOS III average respectively.

Model 1 had quantities of beans, nuts, and seeds comparable to the SOS III average. However, Models 2 & 3 had almost double the consumption compared to the SOS III observed diets. Model 1 had similar fruit quantities to the SOS III average, while Models 2 & 3 increased the amounts of fruits. Finally, the calculated cost for dietary patterns created by the three all three models were comparable to the observed SOS III diet.

The nutrient composition and the cost of the models in comparisons to the SOS III mean diet is next described in Table 7.

The optimized food patterns were more nutrient dense than the observed SOS III diet. First, the modeled food patterns were approximately equicaloric. Second, the amounts of total fat, saturated fat, MUFA, and PUFA were reduced, as were the amounts of total and added sugars and sodium. That was consistent with the imposition of specific constraints on the nutrients to limit. Third, each modeled food pattern was higher in protein, fiber, vitamin A (RAE), vitamin D, vitamin E, vitamin C, thiamin, riboflavin, niacin, calcium, iron, and substantially higher in potassium, as compared to the SOS III mean. The modeled food patterns were slightly lower in zinc and vitamin B-12. Most importantly, all three models were within each of the 22 nutrient constraints defined above.

### 3.4. A Focus on Foods in the Ultra-Processed Category

Table 8 compares the percent composition of energy and nutrients from unprocessed and ultra-processed foods from the SOS III mean and from Model 1. Table A1 shows the full percent splits of energy and nutrients by NOVA food processing categories for all models.

In the SOS III sample and in all the models, more than half of dietary energy came from ultra-processed foods. Since ultra-processed foods were more energy dense, they contributed less than half of the weight of the observed diet and modeled food patterns. As shown by the observed diets and modeled food patterns (Model 1), ultra-processed foods accounted for the bulk of added sugar (65.0%), total sugar (98.5%), sodium (87.1%), carbohydrates (63.4 %) and saturated fat (46.1%). Protein was more evenly distributed among unprocessed and ultra-processed foods in the SOS III observed diets, while in Model 1 it was slightly skewed towards unprocessed foods.

Substantial differences were observed in protein by food source. In the SOS III diets, most of the animal protein (76.5%) and cholesterol (68.3%) came from unprocessed meat, poultry and fish. By contrast, most plant protein came from ultra-processed foods. In addition to saturated fat, ultra-processed foods accounted for the bulk of MUFA and PUFA. The same distributions were observed in the modeled food patterns (Model 1).

Whereas fiber came mostly from ultra-processed foods in the SOS III sample, Model 1 had an equal contribution of fiber from unprocessed and ultra-processed foods.

Although ultra-processed foods were the principal dietary sources of added sugar, sodium and saturated fat, they also provided substantial amounts of vitamin E, thiamin, niacin, folate, and calcium. These micronutrients mostly came from ultra-processed foods both in the observed SOS III diets and in the modeled food patterns.

On the other hand, some vitamins came largely from unprocessed foods. Those included vitamin C, vitamin D, vitamin B-6 and vitamin B-12. That finding held for both the observed SOS III diets and for the modeled food patterns. Notably, in the SOS III, 56.7% of vitamin B-12 came from unprocessed foods; that percentage increased to 84.6% in Model 1. Similarly, vitamin A came mostly from unprocessed foods in SOS III diets, but from ultra-processed foods in modeled food patterns.

Iron came primarily from ultra-processed foods in both the SOS III diets and in modeled food patterns while zinc was more evenly split. Finally, although potassium and water were evenly split in SOS III diets, it came from unprocessed foods in Model 1. For the observed SOS III diets, estimated diet cost was evenly split between unprocessed and ultra-processed foods. In Model 1, ultra-processed foods slightly edged out the unprocessed foods with 53.7% of the cost. In Models 2 & 3, much of the cost came from the ultra-processed foods.

Finally, Table 9 shows the top five foods in each of the food groups ordered by energy for Models 1 and 2 & 3 respectively. Models 2 & 3 contained fewer foods, particularly in the milk and dairy and meat, poultry, and fish groups. In addition, many of the foods contained in this pattern were highly fortified foods. The food pattern from Model 1 contained a more balanced assortment of foods, with high quantities of vegetables, grains, and meats. However, it also contained some fortified ultra-processed foods, such as the Vitamin C fortified drink. The full food patterns for these models are shown in Table A2 and Table A3.

### 3.5. Lowering Vitamin D Requirements

Section 3.2 demonstrated that there were only three market basket conditions in which a LP solution was obtained. An overly high vitamin D requirement in Section 3.2 could have prevented LP solutions from the other models. As such, we reduced the vitamin D requirement by 50%, from 20 mcg to 10 mcg, and kept all other requirements the same. After re-running the models, we obtained Table 10, which shows that model-generated patterns were feasible for all market basket combinations.

Specifically, Table 11 shows the gram and energy distributions split by various categories for the ultra-processed-exclusive food pattern (Model 4) and the unprocessed-exclusive food pattern (Model 5), both with reduced Vitamin D requirements.

Ultra-processed foods had a higher caloric density, as both Models 4 and 5 had 2000 kcal, but Model 4 had only 1327.3 g compared to 2562.8 g in Model 5. Notably, both models contained less fats and sweets than the SOS III mean; Model 5 contained *no* foods in this category. While Model 4 contained less milk and dairy foods than the SOS III mean in grams, it had comparable energy. Similarly, Model 5 had almost double the gram quantity of milk and dairy, but an energy quantity nearly identical to the SOS III mean. Model 5 significantly increased the quantities of vegetables consumed, achieving almost a threefold increase compared to the SOS III mean, while Model 4 decreased the gram quantity of vegetables, while raising the total energy. Both models have similar quantities of beans, nuts, and seeds to the population mean. Model 4 had a higher caloric intake of grains, compared to the population mean, while Model 5 was similar. Finally, Model 4 had a significantly lower amount of meat, poultry, and fish than the SOS III mean, while Model 5 had a slightly higher quantity.

In the processed-only diet (Model 4), slightly more of the protein came from plant sources. For the unprocessed-only diet (Model 5), this was reversed. Finally, Model 4 was close to the population mean of $9.10 and was significantly cheaper than Model 5 with a cost of $9.20, while Model 5 had a cost of $18.48.

Finally, Table 12 compares the specific foods chosen by the ultra-processed-only model (Model 4) and the unprocessed-only model (Model 5). Model 4 contained a large quantity of foods, including a high quantity of potato chips and fortified drinks. Model 5 contained more fresh fruits and vegetables. Both models utilized fish as the primary source of meat, although Model 4 supplemented protein with tofu, while Model 5 used high quantities of fresh fish. Finally, Model 5 contained no fats and sweets. The full food patterns for these models are shown in Table A4 and Table A5.

## 4. Discussion

In this work, we used linear programming to generate nutritionally adequate food patterns similar to diets from a sample of people in three counties in Washington. First, we demonstrated that a combination of unprocessed and ultra-processed foods were required for the model to generate a feasible food pattern. Notably, models consisting of just unprocessed foods or just ultra-processed foods did not have any solutions. This was a surprising result, as ultraprocessed foods have historically been disparaged due to their negative health effects [11]. However, we show they are an essential component of any food pattern that seeks to be nutritionally adequate.

The present analyses clearly show that foods that fall into the NOVA ultra-processed category were the principal sources of added sugar, sodium and saturated fat. In that respect, the present results are consistent with past reports [3,4]. On the other hand, those same foods were also among the major contributors of vitamin E, thiamin, niacin, folate, and calcium, and were the main sources of plant protein. That was due to several factors. First, vitamin E is contained in oils used in food processing, ultra-processed white bread has been enriched with B-vitamins and folic acid, whereas calcium is provided not only by yogurt and pizza, but also by fortified beverages. Plant proteins are provided by grain-based products, most of which fall into the ultra-processed category. The present data point to the need for further studies on the contribution of processed fortified foods to the American diet.

In the linear programming analyses, vitamin D was the limiting nutrient. Lowering vitamin D requirements to 10 mcg allowed for the creation of modeled food patterns from the unprocessed and the ultra-processed market baskets respectively. The present findings that the current vitamin D requirements could not be met by any combination of fresh, unprocessed foods suggests that the NOVA scheme misclassifies dairy products, placing many in the processed and ultra-processed categories. Vitamin D and calcium requirements are met more easily by the inclusion of dairy in the habitual diet. Fortification of processed foods may play an additional role.

Nutritionally adequate food patterns constructed using a market basket of ultra-processed foods were higher in plant proteins than in animal proteins. By contrast, food patterns constructed using unprocessed foods only were higher in animal proteins from meat and fish. This would suggest that the major sources of desirable plant proteins are foods that fall into the ultra-processed category, including extruded grains and legumes. Modeled food patterns from the ultra-processed food market basket were also more energy dense. This was due to vegetables being replaced by grains and fortified beverages. These food patterns were also lower in cost, consistent with past data on the low cost of many ultra-processed foods [10].

Although modeled food patterns based on unprocessed foods only were feasible (see Model 5), such patterns incorporated high amounts of fish and vegetables to meet all nutrient constraints. The amounts of fish and vegetables were significantly above the observed diets of the SOS III cohort. Furthermore, modeled food patterns from unprocessed foods only were prohibitively expensive at a daily cost of $18.48.

The work here has several limitations. First, the SOS III dataset consists primarily of women. The optimized food pattern we generate based on the SOS III sample could be improved by using a more representative sample of men and women. Second, the food patterns we generated were limited to the 360 foods extracted from the FFQ. The FFQ component foods that were included in the models were, of necessity, a very limited proxy for the much broader US food supply. Third, the component foods do not contain indicators of fortification, preventing an investigation of foods by fortification status.

Future research directions should include the application of linear programming models with modified nutrient constraints, as the quality of the modeled food pattern may change significantly based on specific constraints. In addition, food patterns should also be varied by changing the objective function in the linear programming models. In this work, we prioritized developing a food pattern consistent with what was observed in the population we studied. In future work, other methods could be employed, such as changing the objective function to minimize cost or minimize differences between the generated food patterns’ nutrient values and the respective RDAs. In addition, the recent increase in Vitamin D requirements may justify further exploring the role of fortified foods in the American diet. Together, these findings point to the role of food manufacturers and their potential for the reformulation of the nutrient density of the food supply.

## Figures and Tables

**Table 1:** Food processing level definitions.

Food Processing Level	Description
1	unprocessed/minimally processed
2	processed culinary ingredients
3	processed foods
4	ultra-processed foods

**Table 2:** Mean energy and nutrient intakes for the SOS III sample compared to the linear programming model constraints.

Dietary Component	SOS III Sample (n=857)			Linear Program Constraints	
	Mean	Median	Std. Dev.	Min. Value	Max Value
Energy (kcal)	1953	1869	775	2000	2000
Protein (g)	81.9	77.7	34.0	50	100
Saturated fat (g)	23.2	20.9	11.4	0	22
MUFA (g)	26.4	24.0	12.7	10	20
PUFA (g)	17.6	16.2	8.5	10	20
Fiber (g)	22.8	21.7	10.0	28	56
Vitamin A RAE (mcg)	1012	834	803	800	1600
Vitamin D (mcg)	6	5	5	20	40
Vitamin E (mg)	10	9	5	15	30
Vitamin C (mg)	125	108	85	90	180
Thiamin (mg)	1.7	1.6	0.7	1.2	2.4
Riboflavin (mg)	1.9	1.8	0.9	1.3	2.6
Niacin (mg)	23.5	22.1	9.8	16	32
Folate (mcg)	405	382	171	400	800
Vitamin B-12 mg	5.5	4.4	4.7	2.4	4.8
Calcium (mg)	953	863	485	1300	2600
Iron (mg)	14.6	13.7	6.5	18	36
Zinc (mg)	11.5	10.7	5.2	11	22
Sodium (mg)	3403	3162	1467	0	2300
Potassium (mg)	2883	2715	1145	4700	9400
Total sugar (g)	100.7	89.1	52.7	0	90
Added sugar (g)	47.6	39.6	33.7	0	50

**Table 3:** Diet definitions.

Food Processing Levels Included	Diet Description
1	Unprocessed/minimally processed foods
1,2	Unprocessed/minimally processed foods and processed ingredients
1,2,3	All foods *excluding* ultra-processed foods
1,2,3,4	All foods
4,3,2	All foods *excluding* unprocessed/minimally foods
4,3	Ultra-processed and highly processed foods
4	Ultra-processed foods

**Table 4:** SOS III Demographics. Twelve participants were excluded due to caloric intake >5000 kcal. Three participants were excluded due to caloric intake <500 kcal.

	Overall Sample	
	*n*	%
**Total**	857	100
**Age (years)**		
21 to 39	285	33.2
40 to 49	256	29.9
50 to 61	316	36.9
**Gender**		
Men	155	18.1
Women	702	81.9
**Race/ethnicity**		
Non-Hispanic White	403	47.2
Hispanic	361	42.3
Other	90	10.5
Missing	3	-
**Education**		
High school or less	304	35.5
Some college	182	21.2
College graduate/graduate school	371	43.3
**Household income**		
Less than $35,000	283	36.9
$35,000 to less than $75,000	210	27.4
$75,000 or more	274	35.7
Missing	90	-

**Table 5:** Overview of feasibility studies.

Model	NOVA Cat.	1	2	3	4	Solution?
1	1,2,3,4	✓	✓	✓	✓	✓
2	2,3,4	✗	✓	✓	✓	✓
3	3,4	✗	✗	✓	✓	✓
4	4	✗	✗	✗	✓	✗
5	1	✓	✗	✗	✗	✗
6	1,2	✓	✓	✗	✗	✗
7	1,2,3	✓	✓	✓	✗	✗

**Table 6:** Average quantities (g/day and kcal/day) in SOS III vs Models 1 and 2 & 3.

	SOS III Mean (n=857)				Model 1		Model 2 & 3	
	Grams		Kcal		Grams	Kcal	Grams	Kcal
	Mean	Stdev	Mean	Stdev				
**Total**	1679.0	702.7	1953	775	1749.3	2000	1323.5	2000
**Food groups**								
Fats and sweets	296.9	328.1	319.9	235.3	234.6	252.3	242.1	182.5
Milk & dairy	198.7	213.8	176.8	143.7	273.6	176.9	36.6	50.7
Fruits	239.6	221.6	130.4	112.1	239.6	130.4	304.4	130.4
Vegetables	330.9	187.6	154.8	84.9	476.8	397.4	73.5	97.8
Beans, nuts, and seeds	83.2	98.8	132.2	122.4	83.2	133.8	245.5	254.0
Grains	319.8	174.7	666.2	362.5	290.5	629.8	289.1	1022.9
Meat, poultry, and fish	209.8	137.1	372.4	248.2	150.9	279.3	132.2	261.7
**Protein**								
Animal	51.4	26.5	205.6	106.0	48.1	192.3	47.8	191.3
Plant	30.4	14.0	121.6	56.0	42.3	169.2	52.2	208.7
**NOVA categorization**								
Unprocessed	686.6	323.2	581.8	267.4	880.5	765.3	-	-
Culinary ingredients	16.2	15.1	83.7	68.6	5.2	45.9	-	-
Processed	122.2	187.7	101.0	115.1	63.8	86.8	239.6	506.6
Ultra-processed	854.1	509.5	1186.3	566.4	799.8	1102.0	1083.6	1493.4
**Cost ($)**	9.1	3.7	9.1	3.7	7.79		8.04	

**Table 7:** SOS III Means vs Models 1 and 2 & 3.

Dietary Component	SOS III Mean	Model 1	Model 2 & 3
Energy (kcal)	1953	2000	2000
Protein (g)	81.9	90.4	100.0
Saturated fat (g)	23.2	16.70	9.8
MUFA (g)	26.4	19.0	13.3
PUFA (g)	17.6	14.2	15.6
Fiber (g)	22.8	28.0	32.7
Vitamin A RAE (mcg)	1012	1600	1600
Vitamin D (mcg)	5.8	20.0	20.0
Vitamin E (mg)	10.0	23.0	23.4
Vitamin C (mg)	124.8	151.7	162.2
Thiamin (mg)	1.7	1.90	1.7
Riboflavin (mg)	1.9	2.1	1.8
Niacin (mg)	23.5	24.7	32.0
Folate (mcg)	404.5	439.0	400.0
Vitamin B-12 (mg)	5.5	4.8	4.8
Calcium (mg)	953	1300	1300
Iron (mg)	14.6	18.0	18.0
Zinc (mg)	11.5	11.0	11.0
Sodium (mg)	3403	2300	2300
Potassium (mg)	2883	4700	4700
Total sugar (g)	100.7	89.9	75.2
Added sugar (g)	47.6	43.9	31.2
Cost ($)	9.09	7.79	8.0

**Table 8:** Percent composition of nutrients and other variables in the SOS III dataset and in Model 1.

	SOS III Mean		Model 1	
% Composition	Food Proc.		Food Proc.	
	1	4	1	4
**General**				
Energy (kcal)	30.5	59.7	38.3	55.1
Grams	42.1	49.5	50.3	45.7
Cost ($)	45.2	46.1	41.8	53.7
**Macronutrients**				
Total carbs (g)	27.7	65.9	34.3	63.4
Total protein (g)	48.4	48.9	54.1	44.0
Total fat (g)	27.3	59.2	29.9	49.4
**Carbohydrates**				
Total sugar (g)	29.6	61.5	30.4	65.0
Added sugar (g)	3.1	83.4	0.0	98.5
**Protein**				
Animal protein (g)	61.0	38.0	76.5	22.4
Plant protein (g)	26.3	68.1	28.8	68.3
**Fats**				
Cholesterol (mg)	69.1	28.2	71.4	19.3
Saturated fat (g)	28.8	59.9	33.1	46.1
MUFA (g)	29.3	54.5	30.4	42.5
PUFA (g)	18.5	67.9	21.7	63.7
**Micronutrients**				
Sodium (mg)	29.6	68.2	11.6	87.1
Vitamin A RAE (mcg)	62.4	31.6	15.4	82.0
Vitamin D (mcg)	62.2	36.8	62.4	37.0
Vitamin E (mg)	30.9	53.4	6.3	88.3
Vitamin C (mg)	62.7	36.0	53.0	46.6
Thiamin (mg)	30.1	66.7	29.9	68.0
Riboflavin (mg)	42.5	54.2	47.3	49.7
Niacin (mg)	40.3	55.4	45.5	53.8
Vitamin B6 (mg)	51.3	44.0	63.3	35.5
Folate (mcg)	38.9	57.7	42.2	54.9
Vitamin B-12 (mg)	56.7	42.0	84.6	14.5
Calcium (mg)	30.1	67.6	30.8	67.1
Fiber (g)	40.2	54.8	48.3	47.2
Iron (mg)	32.2	63.7	37.7	60.6
Zinc (mg)	46.0	50.9	52.2	45.0
Potassium (mg)	48.8	46.6	68.6	29.4

**Table 9:** Comparison of food patterns generated for Models 1 and 2 & 3 (top five caloric foods in each food group displayed).

Model 1			Models 2 & 3		
Item	Food Proc.	Energy (kcal)	Item	Food Proc.	Energy (kcal)
**Total**	-	2000	**Total**	-	2000
**Fats and sweets**			**Fats and sweets**		
Low/reduced fat mayonnaise	4	54.3	Hot chocolate	4	175.8
Hot chocolate	4	54.0	Canned, liquid Ensure	4	6.7
Snickers bar candy	4	42.3			
Cola soft drink	4	40.5			
Olive oil	2	27.7			
**Milk & dairy**			**Milk & dairy**		
1% Milk	1	94.5	Nonfat, chocolate frozen yogurt	4	50.7
Sour cream	3	37.8			
Nonfat, chocolate frozen yogurt	4	23.7			
Reduced fat cheddar cheese	4	21.0			
**Fruits**			**Fruits**		
Vitamin C fortified drink	4	44.6	Calcium fortified drink	4	95.2
Fresh banana	1	31.00	Apple juice	4	34.6
Applesauce	3	12.5	Tomato juice	4	0.7
Grape juice	4	10.1			
Grapefruit juice	4	6.5			
**Vegetables**			**Vegetables**		
Baked potato w/skin	1	332.9	Mashed potato w/milk & fat	4	37.1
Raw cabbage	1	20.5	Canned corn	3	22.6
Fresh avocado	1	18.8	Commercial french fries	4	19.3
Potato salad w/mayo	4	14.1	Commercial hashbrowns	4	18.8
Ketchup	4	7.2			
**Beans, nuts, and seeds**			**Beans, nuts, and seeds**		
Firm tofu	4	43.4	Firm tofu	4	142.6
Smooth peanut butter	4	38.1	Cooked pinto beans	3	50.1
Mixed nuts w/o peanuts	3	24.0	Low-fat tofu	4	45.8
Lima beans	1	10.8	Baked beans	4	11.7
Baked beans	4	9.0	Canned refried beans	4	2.5
**Grains**			**Grains**		
Plain, white English muffin	4	307.4	Low-fat potato chips	4	573.5
Nonfat potato chips	4	193.6	Nonfat potato chips	4	216.4
Bean & cheese burrito	4	76.1	Nonfat, air-popped popcorn	3	172.2
Granola bar	4	24.8	Cheerios cereal	4	60.9
Cheese & beef enchilada	4	17.6			
**Meat, poultry, and fish**			**Meat, poultry, and fish**		
Roasted chicken thigh w/o skin	1	101.8	Canned, light tuna w/oil	3	261.7
Bluefish	1	61.0			
Roasted pork loin	1	56.7			
Oil-packed, light tuna salad w/mayo	4	26.3			
Low-fat bologna	4	17.1			

**Table 10:** Feasibility studies with reduced vitamin D requirement.

Model	NOVA Cat.	1	2	3	4	Solution?
1	1,2,3,4	✓	✓	✓	✓	✓
2	2,3,4	✗	✓	✓	✓	✓
3	3,4	✗	✗	✓	✓	✓
4	4	✗	✗	✗	✓	✓
5	1	✓	✗	✗	✗	✓
6	1,2	✓	✓	✗	✗	✓
7	1,2,3	✓	✓	✓	✗	✓

**Table 11:** Average quantities (g/day and kcal/day) in SOS III vs. Models 4 & 5 with modified Vitamin D requirements.

	SOS III Mean (*n* = 857)				Model 4 (Reduced Vit. D)		Model 5 (Reduced Vit. D)	
	Grams		Energy (kcal)		Grams	Energy (kcal)	Grams	Energy (kcal)
	Mean	Stdev	Mean	Stdev				
**Total**	1679	702.7	1953	775	1327.3	2000	2562.8	2000
**Food Groups**								
Fats and sweets	296.9	328.1	319.9	235.3	233.1	171.0	0.00	0.00
Milk & dairy	198.7	213.8	176.8	143.7	136.88	183.4	354.8	178.9
Fruits	239.6	221.6	130.4	112.1	253.7	111.4	465.6	212.6
Vegetables	330.9	187.6	154.8	84.9	150.5	257.9	954.8	395.0
Beans, nuts, and seeds	83.2	98.8	132.2	122.4	105.3	134.1	63.7	132.2
Grains	319.8	174.7	666.2	362.5	319.8	967.7	465.9	647.5
Meat, poultry, and fish	209.8	137.1	372.4	248.2	128.0	174.5	258.0	433.8
**Protein**								
Animal	51.4	26.5	205.6	106	33.4	133.5	49.9	199.7
Plant	30.4	14	121.6	56	37.2	148.9	45.0	180.1
**NOVA categorization**								
Unprocessed	686.6	323.2	581.8	267.4	-	-	2562.8	2000
Culinary ingredients	16.2	15.1	83.7	68.6	-	-	-	-
Processed	122.2	187.7	101	115.1	-	-	-	-
Ultra-processed	854.1	509.5	1186.3	566.4	1327.3	2000	-	-
**Cost ($)**	9.1	3.7	9.1	3.7	9.20		18.48	

**Table 12:** Comparison of food patterns generated for Models 4 and 5 (top five caloric foods in each food group displayed).

Model 4 (Reduced Vit. D)		Model 5 (Reduced Vit. D)	
Item	Energy (kcal)	Item	Energy (kcal)
**Total**	2000	**Total**	2000
**Fats and sweets**		**Fats and sweets**	
Hot chocolate	161.2		
Liquid Slim-Fast	6.4		
Hi-C drink	3.4		
**Milk & dairy**		**Milk & dairy**	
Whole milk ricotta cheese	75.1	1% Milk	139.8
Nonfat fruit yogurt	65.2	Cream	32.2
Ice cream shake	23.8	Plain, low-fat yogurt	6.9
Nonfat cheese	19.3		
**Fruits**		**Fruits**	
Vitamin C fortified drink	86.7	Fresh blackberries	193.7
Grape juice	15.2	Grapes	6.99
Grapefruit juice	9.1	Dried apricots	12.0
Tomato juice	0.6		
**Vegetables**		**Vegetables**	
Commercial hashbrowns	204.9	Baked potato w/skin	114.2
Mashed potato w/milk and fat	37.1	Cooked summer squash	94.8
Green pea soup	10.1	Cooked garlic	90.6
V-8 vegetable juice	5.7	Frozen, cooked string beans	48.9
		Fresh avocado	17.0
**Beans, nuts, and seeds**		**Beans, nuts, and seeds**	
Low-fat tofu	81.7	Homemade refried beans	120.9
Smooth peanut butter	30.1	Lima beans	11.3
Canned refried beans	9.9		
Baked beans	5.3		
Soy milk	5.1		
**Grains**		**Grains**	
Low-fat potato chips	600.2	Cornbread (homemeade)	410.0
Nonfat potato chips	136.5	Oatmeal	176.6
Granola	109.2	Cooked pasta	60.8
Cheerios cereal	59.8		
Prepackaged, flavored oatmeal w/water	28.5		
**Meat, poultry, and fish**		**Meat, poultry, and fish**	
Water-packed, light tuna salad w/mayo	132.2	Commercial, fried cod fillets	330.2
Oil-packed, light tuna salad w/mayo	42.3	Non-fried shrimp	103.6

## Data Availability

The datasets generated and/or analyzed during the current study are not publicly available because the data are part of an ongoing study.

## Abbreviations

The following abbreviations are used in this manuscript:

LP	Linear Programming
SOS III	Seattle Obesity Study

## Appendix A

The appendix consists of five tables. Table A1 contains the percent composition of nutrients from the SOS III mean and in Models 1, 2, and 3. The subsequent tables list the foods contained in the patterns generated by different models, including cost, processing level, mass, and energy information stratified by food group. From the initial experimentation, Table A2 and Table A3 describe the food patterns for Model 1 and Models 2 & 3 respectively. Table A4 and Table A5 describe the food patterns for Model 4 and 5 respectively, when the vitamin D requirement is dropped to 10 mcg.

**Table A1:** Percent composition of nutrients and other variables in the SOS III dataset and in the generated models.

	SOS III Mean				Model 1				Model 2 & 3	
% Composition	Food Proc.				Food Proc.				Food Proc.	
	1	2	3	4	1	2	3	4	3	4
**General**										
Energy (kcal)	30.5	4.4	5.4	59.7	38.3	2.3	4.3	55.1	25.3	74.7
Grams	42.1	1.1	7.3	49.5	50.3	0.3	3.6	45.7	18.1	81.9
Cost ($)	45.2	1.3	7.4	46.1	41.8	0.7	3.9	53.7	23.8	76.2
**Overall Macronutrients**										
Total Carbs (g)	27.7	2.1	4.4	65.9	34.3	0.0	2.3	63.4	14.3	85.7
Total Protein (g)	48.4	0.2	2.6	48.9	54.1	0.0	1.9	44.0	48.1	51.9
Total Fat (g)	27.3	10.0	3.5	59.2	29.9	9.6	11.1	49.4	31.6	68.4
**Carbhoydrates**										
Total Sugar (g)	29.6	4.8	4.1	61.5	30.4	0.0	4.6	65.0	2.0	98.0
Added Sugar (g)	3.1	9.3	4.2	83.4	0.0	0.0	1.5	98.5	0.0	100.0
**Proteins**										
Animal Protein (g)	61.0	0.4	0.7	38.0	76.5	0.0	1.1	22.4	80.5	19.5
Plant Protein (g)	26.3	0.0	5.6	68.1	28.8	0.0	2.8	68.3	18.5	81.5
**Fats**										
Cholesterol (mg)	69.1	2.4	0.3	28.2	71.4	0.1	9.2	19.3	55.9	44.1
Saturated fat (g)	28.8	9.4	1.9	59.9	33.1	4.7	16.1	46.1	24.3	75.7
MUFA (g)	29.3	11.8	4.3	54.5	30.4	14.5	12.6	42.5	33.3	66.7
PUFA (g)	18.5	9.3	4.3	67.9	21.7	10.4	4.2	63.7	32.5	67.5
**Micronutrients**										
Sodium (mg)	29.6	0.6	1.7	68.2	11.6	0.0	1.3	87.1	25.4	74.6
Vitamin A RAE (mcg)	62.4	2.4	3.6	31.6	15.4	0.0	2.6	82.0	4.0	96.0
Vitamin D (mcg)	62.2	0.3	0.7	36.8	62.4	0.0	0.7	37.0	39.0	61.0
Vitamin E (mg)	30.9	7.9	7.8	53.4	6.3	3.0	2.4	88.3	6.9	93.1
Vitamin C	62.7	0.1	1.2	36.0	53.0	0.0	0.4	46.6	1.6	98.4
Thiamin (mg)	30.1	0.1	3.1	66.7	29.9	0.0	2.1	68.0	9.9	90.1
Riboflavin (mg)	42.5	0.5	2.8	54.2	47.3	0.0	3.0	49.7	13.1	86.9
Niacin (mg)	40.3	0.0	4.2	55.4	45.5	0.0	0.7	53.8	55.8	44.2
Vitamin B6 (mg)	51.3	0.1	4.6	44.0	63.3	0.0	1.2	35.5	10.6	89.4
Folate (mcg)	38.9	0.1	3.4	57.7	42.2	0.0	3.0	54.9	23.6	76.4
Vitamin B-12 (mg)	56.7	0.3	1.0	42.0	84.6	0.0	0.9	14.5	60.6	39.4
Calcium (mg)	30.1	0.6	1.8	67.6	30.8	0.0	2.1	67.1	2.9	97.1
Fiber (g)	40.2	0.0	5.0	54.8	48.3	0.0	4.4	47.2	31.1	68.9
Iron (mg)	32.2	0.2	3.9	63.7	37.7	0.1	1.5	60.6	23.5	76.5
Zinc (mg)	46.0	0.2	2.9	50.9	52.2	0.0	2.8	45.0	27.4	72.6
Potassium (mg)	48.8	0.3	4.3	46.6	68.6	0.0	2.0	29.4	13.3	86.7

**Table A2:** Food pattern generated for Model 1.

	Model 1			
Item	Cost ($)	Food Proc.	Grams	Energy (kcal)
**Total**	7.79	-	1749.3	2000
**Fats and sweets**				
Low/reduced fat mayonnaise	0.06	4	16.8	54.3
Hot chocolate	0.03	4	72.2	53.9
Snickers bar candy	0.17	4	8.9	42.3
Cola soft drink	0.05	4	109.5	40.5
Olive oil	0.05	2	3.1	27.7
Soybean/cottonseed oil	0.01	2	2.0	17.8
Canned, liquid Ensure	0.06	4	8.9	8.8
Hi-C drink	0.00	4	11.6	5.4
Nonfat mayonnaise	0.01	4	1.6	1.1
Butter	0.00	2	0.1	0.4
**Milk & dairy**				
1% Milk	0.40	1	225.0	94.5
Sour cream	0.07	3	19.4	37.8
Nonfat, chocolate frozen yogurt	0.04	4	17.1	23.7
Reduced fat cheddar cheese	0.15	4	12.1	21.0
**Fruits**				
Vitamin C fortified drink	0.20	4	106.2	44.6
Fresh banana	0.08	1	34.8	31.0
Applesauce	0.11	3	29.1	12.5
Grape juice	0.03	4	16.5	10.1
Grapefruit juice	0.02	4	13.9	6.5
Raisins	0.02	1	2.0	5.9
Fresh apples w/skin	0.04	1	11.1	5.8
Fresh kiwi	0.10	1	9.0	5.5
Canned pears	0.04	3	5.5	4.1
Fresh raspberries	0.16	1	7.3	3.8
Tomato juice	0.01	4	4.2	0.7
**Vegetables**				
Baked potato w/skin	0.39	1	358.0	332.9
Raw cabbage	0.19	1	85.3	20.5
Fresh avocado	0.20	1	11.3	18.8
Potato salad w/mayo	0.04	4	6.5	14.1
Ketchup	0.02	4	7.5	7.2
Tomato soup	0.01	4	4.5	2.8
Commercial salsa	0.02	4	3.9	1.1
**Beans, nuts, and seeds**				
Firm tofu	0.18	4	46.6	43.4
Smooth peanut butter	0.03	4	6.5	38.1
Mixed nuts w/o peanuts	0.07	3	3.9	24.0
Lima beans	0.03	1	10.4	10.8
Baked beans	0.03	4	9.6	9.0
Cooked pinto beans	0.02	3	5.8	8.4
Lentil soup	0.00	4	0.4	0.2
**Grains**				
Plain, white English muffin	0.73	4	135.4	307.4
Nonfat potato chips	1.37	4	73.5	193.6
Bean & cheese burrito	0.21	4	43.8	76.1
Granola bar	0.06	4	6.5	24.8
Cheese and beef enchilada	0.15	4	12.2	17.6
Cream of wheat w/water	0.02	4	19.1	10.4
**Meat, poultry, and fish**				
Roasted chicken thigh w/o skin	0.33	1	47.8	101.8
Bluefish	0.85	1	38.6	61.0
Roasted pork loin	0.34	1	22.5	56.7
Oil-packed, light tuna salad w/mayo	0.31	4	10.8	26.3
Low-fat bologna	0.17	4	13.7	17.1
Homemade beef and beans chilli	0.13	1	17.5	16.5

**Table A3:** Food pattern generated for Models 2 & 3.

Models 2 & 3				
Item	Cost ($)	Food Proc.	Grams	Energy (kcal)
**Total**	8.04	-	1323.3	2000
**Fats and sweets**				
Hot chocolate	0.11	4	235.3	175.8
Canned, liquid Ensure	0.05	4	6.8	6.7
**Milk & dairy**				
Calcium fortified drink	0.38	4	226.6	95.2
Nonfat, chocolate frozen yogurt	0.08	4	36.6	50.7
**Fruits**				
Apple juice	0.10	4	73.5	34.6
Tomato juice	0.01	4	4.3	0.7
**Vegetables**				
Mashed potato w/milk & fat	0.18	4	32.5	37.1
Canned corn	0.08	3	27.9	22.6
Commercial french fries	0.12	4	6.4	19.3
Commercial hashbrowns	0.02	4	6.8	18.8
**Beans, nuts, and seeds**				
Firm tofu	0.60	4	153.1	142.6
Cooked pinto beans	0.12	3	35.0	50.1
Low-fat tofu	0.21	4	40.6	45.8
Baked beans	0.03	4	12.4	11.7
Canned refried beans	0.01	4	2.7	2.5
Meatless chili	0.01	4	1.6	1.3
**Grains**				
Low-fat potato chips	2.60	4	146.0	573.5
Nonfat potato chips	1.53	4	82.2	216.4
Nonfat, air-popped popcorn	0.28	3	44.5	172.2
Cheerios cereal	0.12	4	16.5	60.9
**Meat, poultry, and fish**				
Canned, light tuna w/oil	1.43	3	132.2	261.7

**Table A4:** Food pattern generated for Model 4 with modified vitamin D requirement.

Model 4 (Modified Vitamin D req.)				
Item	Cost ($)	Food Proc.	Grams	Energy (kcal)
**Total**	9.81	-	1327.3	2000
**Fats and sweets**				
Hot chocolate	0.11	4	215.7	161.2
Liquid Slim-Fast	0.06	4	10.2	6.4
Hi-C drink	0.00	4	7.2	3.4
**Milk & dairy**				
Whole milk ricotta cheese	0.30	4	43.2	75.1
Nonfat fruit yogurt	0.15	4	69.3	65.2
Ice cream shake	0.04	4	14.4	23.8
Nonfat cheese	0.14	4	9.9	19.3
**Fruits**				
Vitamin C fortified drink	0.39	4	206.3	86.7
Grape juice	0.04	4	24.8	15.2
Grapefruit juice	0.03	4	19.3	9.1
Tomato juice	0.01	4	3.3	0.6
**Vegetables**				
Commercial hashbrowns	0.20	4	73.9	204.9
Mashed potato w/milk & fat	0.18	4	32.5	37.1
Green pea soup	0.04	4	14.2	10.1
V-8 vegetable juice	0.05	4	30.0	5.7
**Beans, nuts, and seeds**				
Low-fat tofu	0.38	4	72.4	81.7
Smooth peanut butter	0.03	4	5.1	30.1
Canned refried beans	0.03	4	10.6	9.9
Baked beans	0.02	4	5.6	5.3
Soy milk	0.02	4	9.4	5.1
Firm tofu	0.01	4	1.8	1.7
Meatless chili	0.00	4	0.5	0.4
**Grains**				
Low-fat potato chips	2.72	4	152.8	600.2
Nonfat potato chips	0.96	4	51.8	136.5
Granola	0.15	4	24.0	109.2
Cheerios cereal	0.07	4	16.2	59.8
Prepackaged, flavored oatmeal w/water	0.07	4	51.3	28.5
Water-packed, light tuna casserole	0.22	4	20.2	23.4
Total cereal	0.03	4	2.9	9.3
Bean & cheese tostada	0.00	4	0.7	0.9
**Meat, poultry, and fish**				
Water-packed, light tuna salad w/mayo	2.82	4	110.7	132.2
Oil-packed, light tuna salad w/mayo	0.54	4	17.4	42.3

**Table A5:** Food pattern generated for Model 5 with modified vitamin D requirement.

Model 5 (Modified Vitamin D req.)				
Item	Cost ($)	Food Proc.	Grams	Energy (kcal)
**Total**	18.5	-	2562.8	2000
**Fats and sweets**				
**Milk & dairy**				
1% Milk	0.60	1	332.9	139.8
Cream	0.11	1	11.0	32.2
Plain, low-fat yogurt	0.04	1	10.9	6.9
**Fruits**				
Fresh blackberries	8.24	1	450.5	193.7
Dried apricots	0.08	1	5.0	12.0
Grapes	0.07	1	10.1	7.0
**Vegetables**				
Baked potato w/skin	0.14	1	122.8	114.2
Cooked summer squash	2.23	1	474.1	94.8
Cooked garlic	0.57	1	60.8	90.6
Frozen, cooked string beans	0.68	1	174.7	48.9
Fresh avocado	0.18	1	10.2	17.0
Raw tomatoes	0.29	1	90.5	16.3
Dehydrated mashed potatoes	0.01	1	11.5	10.7
Homemade salsa	0.03	1	10.3	2.5
**Beans, nuts, and seeds**				
Homemade refried beans	0.06	1	52.8	120.9
Lima beans	0.03	1	11.0	11.3
**Grains**				
Cornbread (homemeade)	0.30	1	142.6	410.0
Oatmeal	0.14	1	284.8	176.6
Cooked pasta	0.03	1	38.5	60.8
**Meat, poultry, and fish**				
Commercial, fried cod fillets	2.22	1	153.4	330.2
Non-fried shrimp	2.42	1	104.7	103.6
